# Quality, Integrity, and Transparency in Animal Science: The *Animals* Perspective

**DOI:** 10.3390/ani16030403

**Published:** 2026-01-28

**Authors:** Clive J. C. Phillips

**Affiliations:** 1 Sustainability Policy (CUSP) Institute, Curtin University, Kent St., Bentley, Perth 6102, Australia; clive.phillips58@outlook.com or clive.phillips@curtin.edu.au; 2 Institute of Veterinary Medicine and Animal Science, Estonia University of Life Sciences, Kreutzwaldi 1, 51014 Tartu, Estonia

## 1. Introduction: Purpose and Scope

At its core, a scientific journal exists to deliver new and meaningful knowledge to its readers. This mission is supported by two equally critical responsibilities: publishing work without unnecessary delay and safeguarding the journal’s continued reputation [1].

Since its launch in 2011, *Animals* has evolved into a globally recognized journal with a broad disciplinary remit. The journal’s presence in SCIE, Scopus, and PubMed, together with steadily improving citation metrics, underscores its expanding influence within the research community.

This Editorial aims to outline the core principles that guide editorial decision-making at *Animals*. It is intended to support authors, reviewers, and Editors by clarifying expectations at each stage of the publication process, highlighting common pitfalls, and reinforcing the journal’s commitment to scientific quality, ethical responsibility, and accessibility to both the scientific community and the wider public.

## 2. Preliminary Screening: The Importance of the Initial Evaluation

Editorial pre-screening represents the initial stage of the academic peer-review process. The sheer volume of research manuscripts submitted to journals today makes this early editorial decision-making increasingly demanding [2]. It is very necessary, since it serves as a critical quality-control mechanism that safeguards the scientific rigor and scope of the journal. As a broad-ranging journal in animal science, our Editors aim to ensure inclusivity.

The preliminary screening process is often the first point of contact between a manuscript and the journal. At this stage, submissions are assessed not only for the novelty of their scientific content and the validity of the methods used, but also for quality of English, relevance, and suitability within the journal’s broad scope. *Animals* welcomes quantitative, qualitative, and philosophical studies, provided that the methodology is valid and clear, and that the work contributes something new.

Originality is central to this assessment. While tools such as artificial intelligence may summarize existing knowledge, they cannot generate new ideas. Every paper, including reviews, must therefore offer a novel perspective or insight, even if the contribution is modest. Submissions that lack methodological clarity, novelty, or adequate presentation may be returned to authors before peer review to allow essential improvements to be made.

## 3. Scope and Ethical Boundaries

Although the journal maintains an intentionally broad scope, its boundaries are clearly defined. Studies that use animals solely as models for the development of human medical products fall outside the journal’s remit. This reflects a deliberate decision to avoid publishing work that does not primarily advance animal-related knowledge or welfare.

Ethical assessment is fundamental to research involving animals as subjects, including studies of animal behavior and welfare. While institutional ethics review systems are well-established in many regions, their structure and application vary considerably across cultures, institutions, and geographical settings. We try to adopt a universal standard, rather than just relying on existing ethical review, whilst at the same time prioritizing animal-centered considerations [3]. Research in applied ethology and animal welfare presents particular ethical complexities, as it may necessitate the study of animals under conditions where welfare is temporarily compromised. Moreover, commonly employed behavioral methodologies—such as open field tests, social mixing or close observation by researchers—can induce stress or discomfort in the animals involved. These challenges underscore the heightened ethical responsibility of animal welfare researchers, whose work should be ultimately intended to inform practices and interventions that enhance animal well-being [3].

Ethical approval is a critical consideration from the earliest stages of evaluation. Manuscripts must demonstrate that appropriate ethical standards have been met, whether in experimental use of animals, research involving native species, or other sensitive contexts. Where formal ethical approval processes are absent, which is permissible in some jurisdictions, Editors use their informed ethical judgment, considering both utilitarian arguments and societal expectations. Research must not cross lines that the public would find unacceptable and should ideally deliver benefits to animals themselves or to animal populations more broadly.

## 4. Presentation Quality and Responsible Authorship

High standards of written English are essential. Manuscripts should be submitted in near-final form, with a clear structure and careful attention to good use of the English language. Authors are also advised against fragmenting a single, cohesive study into multiple smaller papers, as a single, well-developed manuscript typically offers greater scientific value than several segmented publications.

One of the most common reasons manuscripts are returned to authors from *Animals* is the absence of a Simple Summary, or the submission of one that is insufficiently clear or intelligible to the public. This section must be simple and focused on conveying the study’s purpose and overall findings in terms understandable to non-specialist readers. Hence introductory material describing why the study was done is very necessary, usually at the start of the Simple Summary. As a journal committed to public accessibility, *Animals* depends on this summary to communicate research beyond the academic community, while avoiding overstatement or conclusions that are not fully supported by the evidence. There are generally fewer problems with the Abstract, but it is important that this does not just repeat the Simple Summary; instead, it should provide more detail of the methods used and a description of the results.

## 5. Introduction and Methodology

Within the manuscript, the Introduction should extend beyond just a summary of the existing literature. Authors are expected to clearly articulate the necessity of the study and demonstrate how it addresses a defined gap, limitation, or unresolved issue. Providing appropriate context, rationale, and justification is essential.

The Methods Section must be reported with sufficient detail to enable reproducibility. This includes full transparency when evaluating commercial products or compounds, with clear disclosure of their composition, production, and processing, irrespective of commercial considerations. Reports of the testing of mixtures of compounds that are tested on animals are only of value if it is clear which component is achieving the reported effects. References to “standard protocols” are inadequate unless they are accompanied by detailed descriptions of their application and their effects on the animals involved, including any potential pain, distress, or suffering.

## 6. Statistical Analysis: Clarity over Complexity

Statistical analysis is becoming increasingly sophisticated, and this is broadly welcomed. However, complexity must not come at the expense of clarity. Analyses should be understandable to animal scientists and should not obscure meaning through unnecessary jargon. Editors and reviewers are encouraged to question statistical approaches they do not understand.

Claims of significance must be appropriately framed. Results with probabilities greater than 0.05 should not be presented as definitive, and while trends or tendencies may be noted for values between 0.05 and 0.1, these should not appear in Abstracts, Simple Summaries, or Conclusions. Power analysis is also encouraged to ensure that experiments are appropriately designed, using neither too many nor too few animals.

## 7. Results and Discussion: Interpretation and Insight

Results require careful scrutiny, but so too does the Discussion. While combining results and their discussion may occasionally be justified, most manuscripts benefit from a dedicated Discussion Section. This section should interpret findings, relate them to the existing literature, and explain their implications.

Editors and reviewers should remain alert to excessive self-citation and to inappropriate requests from reviewers to cite their own work. Such practices undermine scientific integrity and should be challenged unless there are exceptional and clearly justified reasons.

## 8. Final Decisions, Corrections, and Retractions

As manuscripts progress toward final decisions, Editors are required to weigh up reviewer feedback alongside the journal’s standards and ethical obligations. It is not necessarily a majority decision; for instance, on occasion, it is necessary to reject papers where only one of three reviewers has recommended rejection. It is often harder to write a detailed critique that justifies rejection than it is to recommend minor improvements. In certain cases, concerns may emerge after publication that necessitate correction or, in more serious instances, retraction. Such situations require careful deliberation to ensure that any action taken is fair, transparent, and consistent with best practices in scholarly publishing. Retractions play a vital role in safeguarding the integrity of the scientific record. They function to correct the literature and to notify readers when published work contains flaws or errors that undermine the reliability of its findings [4].

## 9. Concluding Remarks

The principles outlined in this Editorial reflect the journal’s commitment to publishing research that is scientifically rigorous, ethically sound, and clearly communicated. From preliminary screening to final editorial decisions, each stage of the process plays a critical role in safeguarding the quality and credibility of the work published in *Animals*.

Authors are encouraged to view these standards not as obstacles but as essential components of responsible scientific reporting. Clear presentation, transparent methodology, thoughtful interpretation of results, and adherence to ethical norms strengthen both individual manuscripts and the discipline as a whole. Reviewers and Editors, in turn, carry a shared responsibility to apply these principles consistently, critically, and fairly.

By maintaining high expectations for originality, ethics, and accessibility, *Animals* seeks to serve not only the scientific community but also the wider public, for whom animal research often carries profound significance. Through collective commitment to these values, the journal will continue to advance animal science in a manner that is trustworthy, impactful, and aligned with societal expectations.
